# Hyperbaric oxygen therapy for late radiation-induced tissue toxicity: prospectively patient-reported outcome measures in breast cancer patients

**DOI:** 10.1186/s13014-016-0700-0

**Published:** 2016-09-29

**Authors:** David N. Teguh, René Bol Raap, Henk Struikmans, Cees Verhoef, Linetta B. Koppert, Arne Koole, Yadi Huang, Rob A. van Hulst

**Affiliations:** 1 Hyperbaar Geneeskundig Centrum Rijswijk, Treubstraat 5a, 2288 EG Rijswijk, The Netherlands; 2 Department of Surgery/Hyperbaric Medicine, Academic Medical Center, University of Amsterdam, Treubstraat 5a, 2288 EG Rijswijk, The Netherlands; 3 Department of Radiation Oncology, Leiden University Medical Centre, Leiden, The Netherlands; 4 Radiotherapy Centre West, Medical Centre Haaglanden, The Hague, The Netherlands; 5 Department of Surgical Oncology, Erasmus MC Cancer Institute, Rotterdam, The Netherlands; 6 Department of Anesthesiology/Hyperbaric Medicine, Academic Medical Center, University of Amsterdam, Treubstraat 5a, 2288 EG Rijswijk, The Netherlands; 7 University of Leuven, Treubstraat 5a, 2288 EG Rijswijk, The Netherlands

**Keywords:** Breast cancer, Radiotherapy, Radiation toxicity, Fibrosis, Pain

## Abstract

**Introduction:**

This study examines patient reported outcome measures of women undergoing hyperbaric oxygen treatment (HBOT) after breast-conserving therapy.

**Method:**

Included were 57 women treated with HBOT for late radiation-induced tissue toxicity (LRITT) referred in the period January 2014-December 2015. HBOT consisted of (on average) 47 sessions. In total, 80 min of 100 % O_2_ was administered under increased pressure of 2.4 ATA. Quality of life was assessed before and after treatment using the European Organization for Research and Treatment of Cancer (EORTC) QLQ-BR23, and a NRS pain score.

**Results:**

Fifty-seven women were available for evaluation before and after treatment. Before HBOT, patients had severe complaints of pain in the arm/shoulder (46 %), swollen arm/hand (14 %), difficulty to raise arm or move it sideways (45 %), pain in the area of the affected breast (67 %), swollen area of the affected breast (45 %), oversensitivity of the affected breast (54 %), and skin problems on/in the area of the affected breast (32 %); post HBOT, severe complaints were still experienced in 17, 7, 22, 15, 13, 15, and 11 % of the women, respectively. Differences were all significant. The NRS pain score improved at least 1 point (range 0–10) in 81 % of the patients (*p* < 0.05).

**Conclusion:**

In these breast cancer patients treated with HBOT for LRITT, the patient-reported outcomes were positive and improvements were observed. HBOT was a well-tolerated treatment for LRITT and its side-effects were both minimal and reversible.

## Introduction

In the Netherlands, around 14,000 women are diagnosed each year with invasive breast cancer; in addition about 1,900 women are diagnosed with a ductal carcinoma in situ [www.oncoline.nl]. According to the GLOBOCAN series of the International Agency for Research on Cancer, one of the most commonly diagnosed cancers worldwide is breast cancer (1.67 million) [1]. Among women in the Netherlands, the cumulative lifetime breast cancer risk is 12–13 % [www.oncoline.nl]. Early detection (particularly via national breast cancer screening and improved systemic therapy) is the main factor to improve breast cancer prognosis [www.oncoline.nl]. In ≥ 60 % of patients, breast-conserving surgery is applied followed by radiotherapy. A boost dose is added in patients with a high risk of developing a true local recurrence.

When radiation is used to treat cancer it also (partly) affects a variety of critical surrounding normal tissues which can become hypocellular, hypovascular and hypoxic, frequently referred to as ‘3 H tissue’ [2]. The hypoxic status of tissues can be counteracted to some extent by oxygenation of normal cells with hyperbaric oxygen therapy (HBOT). The effects of hyperbaric oxygen can be summarized as follows: short-term effects include reduction of edema and phagocytosis activation, and anti-inflammatory effects. Long-term effects include neovascularization, osteoneogenesis, and stimulation of collagen formation by fibroblasts [3]. HBOT has shown beneficial effects in hypoxic diabetic ulcers that result in severe wound-healing problems and osteoradionecrosis [4], and is frequently used for necrotic soft tissues and bone that fails to heal. HBOT also induces significant angiogenesis, which in one study was measurable after eight HBOT sessions [5]. In addition, a significant increase in mobilization of stem cells from the bone marrow occurs when HBOT is applied [6, 7], with wound healing and recovery of normal-tissue radiation injury as the end result [8–10].

In 25–33 %, breast cancer patients experiencing pain, fatigue, sexual problems, anxiety, and depression [11–13]. Fatigue, worsened physical functioning, disturbed body image and lower quality of life scores are the most frequently reported complaints after radiotherapy and chemotherapy [14]. Late radiation-induced tissue toxicity (LRITT) has consequences for aftercare: for example, radiofibrosis influences arm movement and increases the risk of lymphedema [15]. It is also reported from the EORTC 22881–10882 trial that the 10-year risk of developing moderate or severe fibrosis is 26.2 % if a radiation boost is administered (16 Gy) compared to 12.6 % in the no-boost arm [15]. Other effects of LRITT include impaired cosmetic results, fibrosis of the breast, and thoracic wall pain. Fibrosis of the irradiated breast was shown to increase for up to 9 years after treatment [16]. Radiation-induced thoracic wall pain occurs in 9.5–14.9 % of patients at 3-year follow-up [17]. Administering a boost dose further increases the negative impact on both health-related quality of life and cosmetic outcome [17]. In the Dutch Guideline for Breast Cancer (issued in 2002; revised in 2008 and 2012) HBOT is not yet mentioned due to lack of evidence regarding its efficacy in the case of LRITT [www.oncoline.nl].

More than 100 internationally recognized specialist breast cancer researchers, clinicians and healthcare professionals addressed nine thematic areas in summary papers and concluded that there is a need to incorporate standardized patient-reported outcome measures (PROMs) both in clinical trials and in everyday clinical practice [18]. Deterioration in the quality of life of women treated for breast cancer has a considerable and long-term impact on their everyday functioning [19]. To our knowledge no data are available regarding PROMs in patients referred for HBOT due to LRITT. Therefore, this study aimed to use PROMs before and after HBOT to examine whether HBOT can significantly reduce LRITT after breast-conserving therapy.

## Methods

All patients referred to our center (January 2014 through December 2015) for HBOT due to LRITT were asked to participate in this study. Participation consisted of successively completing the following PROMs before and after HBOT: 1) the European Organization for Research and Treatment of Cancer (EORTC) core Quality of Life Questionnaire (QLQ), i.e. the QLQ-C30, 2) the disease-specific EORTC QLQ-BR23, and 3) the EQ-5D health status questionnaire and a numeric rating scale (NRS) pain score.

### Ethics approval and consent to participate

According to the AMC Ethics Committee, no ethical approval or patient consent were needed since late radiation toxicity is a treatment indication for hyperbaric oxygen treatment and the questionnaires were distributed as part of the regular treatment evaluation.

### Hyperbaric oxygen therapy

HBOT treatment consisted of (on average) 47 sessions (1 session a day/5 days a week) in a multiplace (20-person) hyperbaric chamber. In total 80 min of 100 % oxygen was administered to patients under increased pressure of 2.4 atmospheres absolute (ATA) during a 110-min hyperbaric session. At this pressure, 100 % oxygen was delivered via an oronasal mask in four episodes of 20 min, each interrupted by 5 min of air breathing. During pressure changes, great care was taken to avoid barotraumas, particularly of the middle ear, which is the most common side-effect of a hyperbaric treatment [20].

### Quality of life questionnaires

Questionnaires were given at the start of the treatment and in the last week of HBOT. The core questionnaire (QLQ-C30) applies to all patients with cancer, and the disease-specific questionnaire (QLQ-BR23) is designed specifically for patients with breast cancer. The QLQ-C30 includes 30 questions comprising both multi-item scales and single-item measures. The QLQ-BR23 comprises 23 questions incorporating items covering symptoms and side-effects related to different treatment modalities, body image, sexuality, and future perspective. For these items and scales, higher scores indicate more problems. For the QLQ-BR23, scores were dichotomized into no problems (score 1–2) and severe problems (score 3–4). In addition, a NRS (range 0–10) was used to rate pain before and after HBOT. Regarding the EQ-5D, a positive difference of at least 1 point has been noted as improvement.

### Statistical analysis

Analyses were performed in MS Excel and R (http://www.R-project.org)*.* The cumulative linked mixed model was used to compare scores before and after HBOT for the QLQ-BR23, the EQ-5D questionnaires and the NRS pain score. A two-sided p-value of 5 % was considered to be statistically significant.

## Results

A total of 57 female patients with pre- and post HBOT questionnaires (the QLQ-C30 and the QLQ-BR23) were available for evaluation. Table 1 presents the baseline characteristics of the study population.

**Table 1:** Characteristics of the study population (*n* = 57)

Age in years: mean	59
Median (range)	58 (32–78)
T Stage	
Tis	2
T1	23
T2	18
T3	1
T4	1
Unknown	12
N Stage	
N0	29
N1	10
N2	4
N3	1
Unknown	13
Time since radiotherapy (months)	
Mean	57
Median (range)	33 (9–251)
Maximum radiotherapy dose (Gy)	
Mean	53
Median (range)	56 (19–56)
Chemotherapy	
Yes	29
No	27
Unknown	1
Surgery	
Yes	50
No	6
Unknown	1

Before HBOT, 46, 14, 45, 67, 45, 54 and 32 % of the patients had severe complaints of pain in the arm/shoulder, swollen arm/hand, difficulty to raise the arm or to move it sideways, pain in the area of affected breast, swollen area of affected breast, oversensitivity of the affected breast, and skin problems on or in the area of the affected breast, respectively. Post HBOT, the percentages of patients with these severe complaints had decreased (Table 2). Table 2 also shows the results of the cumulative linked mixed model analysis for the relevant questions regarding the side-effects of breast cancer irradiation. All cumulative linked mixed model coefficients were positive and significant.

**Table 2:** Scores on the EORTC BR23 Questionnaire

	n	Pre HBOT, Severe problems	Post HBOT, Severe problems	Improvement	Mean Pre HBOT to Post HBOT	Cumulative linked mixed model (*p* value)
Did you have any pain in your arm or shoulder?	56	46.4 %	16.7 %	42.6 %	2.4 to1.8	1.7 (*p* <0.05)
Did you have a swollen arm or hand?	56	14.3 %	7.4 %	25,9 %	1.6 to 1.4	1.2 (*p* <0.05)
Was it difficult to raise your arm or to move it sideways?	56	44.6 %	22.2 %	42.9 %	2.4 to 1.9	2.1 (*p* <0.05)
Have you had any pain in the area of your affected breast?	56	66.7 %	14.5 %	63.6 %	2.8 to 2.1	2.4 (*p* <0.05)
Was the area of your affected breast swollen?	57	44.6 %	12.7 %	57.9 %	2.4 to 1.7	2.0 (*p* <0.05)
Was the area of your affected breast oversensitive?	57	54.4 %	14.5 %	54.5 %	2.7 to 2.0	1.9 (*p* <0.05)
Have you had skin problems on or in the area of your affected breast (e.g., itchy, dry, flaky)?	57	32.1 %	11.3 %	41.5 %	2.0 to 1.6	1.5 (*p* <0.05)

For the EQ-5D, 46 completed questionnaires could be analyzed. Mobility, self-care, activity, pain, and anxiety improved by 88, 50, 50, 75, and 80 %, respectively. The total EQ-5D score improved in 71 % (median 9.5 to 7.7 points) of the patients while the NRS pain score (median 5 to 2) improved in 81 % of the patients (<0.05).

The side-effects of HBOT were minimal, i.e. 8/57 patients had reversible myopia and 8/57 had reversible tiredness.

## Discussion

This is the first study to show that breast cancer patients treated with HBOT due to LRITT significantly improved on all domains of the QLQ-BR23. In addition, the NRS score improved in the majority of these patients.

LRITT is observed in ≤ 30 % of patients treated for breast cancer [15, 21, 22]. This rate is much higher than the rate of referrals for treatment of these side-effects with hyperbaric oxygen in the clinic. Serious side-effects observed after breast irradiation include impaired cosmetic results, fibrosis of the breast, and thoracic wall pain; unfortunately, for the present study population no data are available on the cosmetic results. Collette et al. published a breast fibrosis nomogram showing a strong association between radiotherapy dose and fibrosis, with large boost volumes as a prognostic factor on univariate analysis only in the EORTC 22881–10882 trials [15]. Carl et al. reported on 44 patients with persisting symptoms after breast-conservation therapy: of these, 32 women received HBOT for a median of 25 sessions and 12 were control patients [23]. The HBOT patients showed a significant reduction in pain, edema and erythema scores using the LENT-SOMA scale (provider reported) as compared to non-HBOT controls; in that study, fibrosis and telangiectasia were not significantly affected by HBOT [23]. In their study, Mukesh et al. examined the volume effect by developing a predictive Normal Tissue Complication Probability (NTCP) model [5] and suggested that the maximum radiotherapy dose is the most important parameter to influence late breast fibrosis [24, 25]. However, the authors warned that this may reflect limitations in the current scoring system, that other radiotherapy-associated complications should also be analyzed to determine the effects of dose-volume parameters, and that PROMs should complement clinician score-based models in the future. Therefore, the authors conclude that inclusion of other clinical factors is recommended for future NTCP modeling work [24, 25].

Vilholm et al. defined the following risk factors for the development of postmastectomy pain syndrome (PMPS): having undergone breast surgery earlier (OR 8.12), tumor located in the upper lateral quarter (OR 6.48), young age (OR 1.04), and axillary lymph node dissection (OR 1.99) [14]. The authors concluded that, although advances in diagnostic and surgical procedures have reduced the frequency of the more invasive surgical procedures, there is still a considerable risk of developing PMPS after treatment for breast cancer, and that development of preventive measures (as well as treatment of the syndrome) are highly relevant [14]. This might be a rationale for starting a prospective study with HBOT immediately followed by radiotherapy in a prophylactic setting.

Limitation in arm movement is another side-effect of breast cancer treatments. Kootstra et al. reported that axillary node dissection affects muscle strength, range of shoulder motion and arm volume [26].

In the Cambridge Breast Intensity Modulated Radiotherapy Trial, Barnett et al. reported patient and treatment-related factors that influence late toxicity, where the greatest risk factors for the development of late toxicity 2 years after breast-conserving surgery and adjuvant breast radiotherapy are a larger breast volume independent of dose inhomogeneity, baseline pre-radiotherapy surgical cosmesis, postoperative infection, and smoking [27]. Previous factors seem to be more important than dose inhomogeneity and the addition of boost radiotherapy at 2 years after the completion of radiotherapy. Unfortunately data on these factors were not available for our patients, which is a limitation of the present study. Mukesh et al. reported that skin telangiectasia is associated with older age, postoperative breast infection and tumor bed boost, and increasing breast volume [28].

No prospectively randomized trials are available on the efficacy of HBOT in breast cancer patients. In a Cochrane review, Bennett et al. concluded that there is some evidence that HBOT improves outcomes in late radiation induced toxicity [4]. In their large double-blind randomized study, Clarke et al. showed the substantial benefit of HBOT on quality of life in chronic refractory radiation induced proctitis [29]. Glover et al. reported in a clinical trial of 46 participants in the hyperbaric oxygen group and 23 participants in the control group, no significant differences in the change of IBDQ bowel component score. From the 29 participants in the hyperbaric oxygen group and 11 participants in the sham group with rectal bleeding at baseline, also no significant differences in the change of IBDQ rectal bleeding score were seen. Several letter to the editors are published regarding the study setup thereafter in the journal [30]. The possible benefit of HBOT immediately after radiotherapy to prevent/reduce late side-effects of radiation in head and neck cancer was examined in another randomized pilot study [2]; this latter study showed that patients receiving hyperbaric oxygen after radiotherapy had better quality of life scores for swallowing, sticky saliva, xerostomia, and pain in the mouth. Tahir et al. presented Australia’s largest study for chronic radiation-induced tissue injuries treated with hyperbaric oxygen, evaluated by the Common Terminology Criteria for Adverse Events v3.0 (CTCAE) grading system [20]. This is a provider-reported study (whereas our study reflects the patient’s point of view) in which the authors demonstrate that HBOT can be effectively applied in a variety of chronic radiation-induced tissue, including the breast.

Our HBOT protocol for LRITT of breast-conserving therapy included (on average) 47 sessions; this number was required for a maximum effect in reducing complaints. Although our protocol is usually patient-tailored for a minimum of 40 sessions, the prescribed amount of treatment varies per individual patient. Future research could examine the optimal amount of HBO treatment to reduce problems related to LRITT. The present study demonstrates a significant improvement in quality of life based on PROMs after HBOT for LRITT after breast-conserving treatment. A randomized controlled trial (including PROMs) is recommended to provide more substantial evidence concerning the value of HBOT in these patients.

## Conclusions

The use of PROMs in breast cancer patients treated with HBOT for radiation-induced late tissue toxicity was valuable and revealed a significant improvement in the related complaints. The side-effects of HBOT were both minimal and reversible.

## Abbreviations

CTCAE: Common Terminology Criteria for Adverse Events
EORTC: European Organization for Research and Treatment of Cancer
HBOT: Hyperbaric oxygen treatment
LRITT: Late radiation-induced tissue toxicity
PROMs: Patient-reported outcome measures
QLQ: Quality of Life Questionnaire
